# Registration Combining Wide and Narrow Baseline Feature Tracking Techniques for Markerless AR Systems

**DOI:** 10.3390/s91210097

**Published:** 2009-12-11

**Authors:** Liya Duan, Tao Guan, Bo Yang

**Affiliations:** Digital Engineering and Simulation Centre, Huazhong University of Science and Technology, No.1037 Luoyu Road, 430074 Wuhan, China; E-Mail: jessduanjessduan@126.com

**Keywords:** augmented reality, registration, natural features, wide baseline, narrow baseline, scale invariant feature transform

## Abstract

Augmented reality (AR) is a field of computer research which deals with the combination of real world and computer generated data. Registration is one of the most difficult problems currently limiting the usability of AR systems. In this paper, we propose a novel natural feature tracking based registration method for AR applications. The proposed method has following advantages: (1) it is simple and efficient, as no man-made markers are needed for both indoor and outdoor AR applications; moreover, it can work with arbitrary geometric shapes including planar, near planar and non planar structures which really enhance the usability of AR systems. (2) Thanks to the reduced SIFT based augmented optical flow tracker, the virtual scene can still be augmented on the specified areas even under the circumstances of occlusion and large changes in viewpoint during the entire process. (3) It is easy to use, because the adaptive classification tree based matching strategy can give us fast and accurate initialization, even when the initial camera is different from the reference image to a large degree. Experimental evaluations validate the performance of the proposed method for online pose tracking and augmentation.

## 1. Introduction

The main intention of augmented reality is to superimpose extra perceptible elements on a user's real world environment, for the purpose of improved understanding and interaction. Since vision plays an important role in human perception, most AR research is concerned with the use of live video imagery which is digitally processed and “augmented” by the addition of computer generated graphics. Thus, AR requires accurate registration of virtual objects in 3D in order to render a virtual object into the real world.

## 2. Related Researches

Many efforts have been carried out on issues relating to track camera pose for markerless AR registration. The method using natural feature points has attracted attention recently. Using feature points of the scene is a very significant approach and gives effectively restricted conditions, because a lot of points exist in indoor or urban environments. According to the points tracking strategy, this kind of work can be divided into two catalogues: narrow baseline and wide baseline registration.

In the first catalogue, different kinds of narrow baseline tracking methods [1-3] are used to establish the feature correspondences between frames. Simon *et al.* [4,5] proposed a registration method using planar structures in the scenes. To overcome the problem of losing features, the Harris corner detector is used to find the features in each input image, and an optical flow tracker is used to track the detected features between successive frames. The registration matrices are computed with the homographies calculated from the obtained feature correspondences. Unfortunately, the method suffers from the problem of error accumulation, and moreover, a reference plane must be specified and other planes need to be perpendicular to this plane under the multiple planes condition. Li *et al.* [6] introduced a registration method for AR based on online estimation of trifocal tensors. A statistical method based on the so-called x84 rule is implemented to remove outliers and normalized cross-correlation (NCC) is used to recover the lost features during tracking process. However, cross-correlation has the drawback that detection is not invariant to viewing direction. Although local predictive warping can alleviate the problem, such methods are always likely to be of limited utility. The above problem also weakens the usability of some top-down methods [7-12]. Yuan *et al.* [13-15] proposed a registration method based on the projective reconstruction technique and the KLT tracker for markerless AR systems. Although the KLT tracker is a useful natural feature tracking method, there are limitations. For example, the camera cannot move rapidly and abruptly when using the KLT tracker. If the camera moves abruptly and rapidly, all the features may be lost and the system will fail. Moreover, these methods don't consider tracking the feature points robustly and is prone to being disturbed by mismatches. Therefore, the registration may be invalidated under the circumstances of large changes in illumination and viewpoint. The similar problems can be seen in [16-19]. Most recently, [20] gives an augmented optical flow tracker based system which is most notable for the evident high-quality patch tracking. It uses a high DOF minimization technique across multiple scales, yielding convincingly better patch tracking results than the NCC search often used in registration. However, it is also computationally expensive and needs user-supplied CAD models to initialize the first frame. The CAD models are not always readily available, and their use is limited to objects that can be easily modeled by hand.

There are mainly two difficulties when using narrow baseline tracking methods for AR systems. The first one is the initialization problem. Since narrow baseline tracking methods do not provide any descriptor about feature points, we cannot automatically determine the initial position of the needed features for the first frame. The second disadvantage is the feature loss problem. This is especially true in the case of features going out of the field of view or occluded by users or some scene objects. Thus the valid matches will become less and less during tracking process, which will finally result in the registration failure.

To overcome the above problems, we can make use of wide baseline matching strategy which works in a tracking by detection style. In the field of object recognition, much research has been conducted into feature points-based object descriptions and several interest point detectors aiming at reliable wide baseline feature matching have been proposed [21-25]. Recently, a comparative study has been carried out by [26], and the scale invariant feature transform, also known as SIFT [23], has been identified as one of the best feature detectors. SIFT is relatively invariant to illumination and viewpoint changes, and is a good candidate for developing model–based tracking. In [27], SIFT features are used for establishing point correspondences between the input frame and those lying on a model which has to be built offline. In [28], a reduced SIFT is implemented on a camera phone for mobile AR purpose. Another very effective wide baseline matching approach is to treat matching as a classification problem. Lepetit and Fua [29,30] adopted random trees and ferns with simple image tests to detect re-occurrences of previously trained keypoint patches in a new input frame. The method can detect re-occurrences even in the case of image noise, changes in scale, rotations, aspect ratio and illumination changes. The classification approach allows the use of a simple classifier which can be carried out quickly. Wagner [28] also implements the random ferns to fulfill the task of feature matching for mobile AR use.

However, while overcoming the problems of initialization and features loss in the narrow baseline method, there are some inherent shortcomings in AR systems based on wide baseline matching techniques. The first one is the problem of frame loss, since there may be a large fraction of outliers in the feature correspondences set, so we are not guaranteed to find a correct pose after a certain random sampling time and have to turn to the next frame to keep the continuity of the system. The second disadvantage is the jittering of the virtual object in the video sequence. This is particularly noticeable when the camera is fully or nearly stationary. This inaccuracy can be a result of image noise, as well as too few or unevenly distributed feature matches.

From the above discussion, we can see that both the wide and narrow baseline tracking strategies have their own limitations respectively. In fact, these two kinds of methods are complements to each other to some degree. So, in this research, we propose a novel feature points tracking strategy combing the wide and narrow baseline techniques to improve the performance of AR systems. Our registration method distinguishes itself in following ways:

1. The method needs no man-made markers for both indoor and outdoor AR applications and can work with arbitrary geometric shapes including planar, near planar and non planar structures which really enhance the usability of AR systems.
2. To initialize the system, we use adaptive classification tree based matching strategy which can provide fast and accurate initialization even when the initial camera is different from the reference image to a large degree.
3. Due to the reduced SIFT based augmented optical flow tracker, the virtual objects can still be augmented on the specified areas even under the circumstances of occlusion and large changes in viewpoint during the online process.

The paper is organized as follows: Section 3 illustrates the scene reconstruction technique. Section 4 presents the proposed natural feature tracking method. Section 5 presents in detail the registration algorithm. Section 6 shows some experimental results. Finally, conclusions are given in the last section.

## 3. Scene Reconstruction Using Direct Bundle Adjustment

The goal of scene reconstruction is to calculate the positions of the 3D points in the real world. The traditional methods are to use five, six or seven-point methods to recover the two-view epipolar constraints between two reference frames. SVD and a check-back step are then taken to get the relative positions between the two reference cameras. Triangulation and bundle adjustment are finally used to obtain the 3D positions of the matched features. The disadvantage of the above methods is that the camera's intrinsic parameters are commonly needed to be determined in advance, once these parameters changed, the calibration is needed to be repeated which really weaken the usability of the AR systems. While self-calibration techniques [31,32] can overcome the above problem to some degree, these techniques cannot effectively cope with missing correspondences and accumulated errors. We make use of an alternative approach suggested in [33,34], which omits the linear initialization step and calculates all the unknown parameters iteratively. We denote the relationship between a 3D point X = (*X,Y,Z*,1)*^T^* and its 2D projection x = (*x,y*,1)*^T^* as follows:

(1) x=λC[R∣t]X=PX

We model each camera using seven parameters, *i.e.*, the rotation expressed by three Euler angles θ = [*θ_x_, θ_y_, θ_z_*], the translation t = [*t_x_, t_y_, t_z_*], and the focal length *f*. The intrinsic matrix is then:

$$\text{C}=\left[\begin{array}{ccc} f & 0 & 0\\ 0 & f & 0\\ 0 & 0 & 1 \end{array}\right]$$

We take a robust sum squared projection error as the objective function. Each matched feature is projected into the reference images, and the sum of squared image distances is minimized with respect to the camera parameters.

Given a measured feature ${\text{x}}_{i}^{k}$, the residual is:

(2) $$\varepsilon_{i}^{k}={\text{x}}_{i}^{k}-{\text{p}}_{i}^{k}$$

where ${\text{p}}_{i}^{k}$ is the projection of x*^k^* in image *i*:

(3) $${\tilde{\text{p}}}_{i}^{k}=\text{C}{\left[\text{R}|\text{t}\right]}_{i}{\text{X}}^{k}$$

The error function is the sum over all images of the residual errors:

(4) $$\text{error}=\sum_{i=1}^{m}\sum_{j=1}^{n}h{(\varepsilon_{i}^{k})}^{2}$$

where *n* is the number of the features, *m* is the number of reference frames. *h*(*x*) is the robust error function:

(5) $$h(x)=\{\begin{array}{ll} {\left|x\right|}^{2}, & \text{if}\left|x\right|<\sigma \\ \sigma , & \text{if}\left|x\right|\geq \sigma \end{array}$$

The error function combines the fast convergence properties of an *L*_2_ norm optimization scheme for inliers (distance less than *σ*), with the robustness of an *L*_1_ norm scheme for outliers (distance greater than *σ*). We use an outlier distance *σ* = ∞ for initialization and *σ* = 1.5 pixels for the final solution.

We cope with the above non-linear least squares problem using the Levenberg-Marquardt algorithm. The form of each iteration step is as follows:

(6) $$\Phi ={\left({\text{J}}^{T}\text{J}+\beta {\text{C}}_{p}^{-1}\right)}^{-1}\;{\text{J}}^{T}\varepsilon$$

where Φ is the vector of all the parameters, ε is the vector of residuals and J = ∂ε/∂Φ. The Jacobean J is an *M* × *N* matrix, where *M* is the number of measurements (twice the number of features), and *N* = *n*_c_ + *n_X_* is the number of camera (*n*_c_) and structure (*n_X_*) parameters (7 for each camera plus 3 for each 3D point). The prior covariance matrix C*_p_* is set such that the standard deviation of angles are *σ_θx_* = *σ_θy_* = *σ_θz_* = π/16, translations *σ_tx_* = *σ_ty_* = *σ_tz_* = 0.005, focal lengths *σ* = *f̄*/50 and 3D points *σ_X_* = *σ_Y_* = *σ_Z_* = 0.05. This helps in choosing suitable step sizes, and hence speeding up convergence. Finally, the parameter *β* is varied in each iteration step to ensure that the objective function of Equation 4 does in fact decrease.

The derivatives are computed analytically via the chain rule, for example:

(7) $$\frac{\partial {\text{p}}_{i}^{k}}{\partial \theta_{ix}}=\frac{\partial {\text{p}}_{i}^{k}}{\partial {\tilde{\text{p}}}_{i}^{k}}\frac{\partial {\tilde{\text{p}}}_{i}^{k}}{\partial \theta_{ix}}$$

where:

(8) $$\frac{\partial {\text{p}}_{i}^{k}}{\partial {\tilde{\text{p}}}_{i}^{k}}=\frac{\partial \left[x/z\;y/z\right]}{\partial \left[x\;y\;z\right]}=\left[\begin{array}{ccc} 1/z & 0 & -x/z^{2}\\ 0 & 1/z & -y/z^{2} \end{array}\right]$$

and:

(9) $$\frac{\partial {\tilde{\text{p}}}_{i}^{k}}{\partial \theta_{ix}}={\text{C}}_{i}\frac{\partial {\text{R}}_{i}}{\partial \theta_{ix}}{\text{X}}^{k},\;{\text{R}}_{i}=e^{{\left[\theta_{i}\right]}_{\times }},\;{\left[\theta_{i}\right]}_{\times }=\left[\begin{array}{ccc} 0 & -\theta_{iz} & \theta_{iy}\\ \theta_{iz} & 0 & -\theta_{ix}\\ -\theta_{iy} & \theta_{ix} & 0 \end{array}\right]$$

(10) $$\frac{\partial {\text{R}}_{i}}{\partial \theta_{ix}}=\frac{\partial }{\partial \theta_{ix}}e^{{\left[\theta_{i}\right]}_{\times }}=e^{{\left[\theta_{i}\right]}_{\times }}\left[\begin{array}{ccc} 0 & 0 & 0\\ 0 & 0 & -1\\ 0 & 1 & 0 \end{array}\right]$$

Instead of solving the Equation 6 directly, we use sparse bundle adjustment [35] to reduce the total computational cost for one step from *O*(*MN*^2^) to $O(mn_{c}^{2})$, where *m* is the number of residuals in each image. Since the number of camera parameters *n_c_* is much less than the number of structure parameters *n_X_*, the above reduction is very significant in practice. For example, with two cameras (*n_c_* = 14), and 100 3D points (*n_X_* = 300), sparse bundle adjustment will be about ((*n_c_* + *n_X_*)/*n_c_* = (314/14)^2^ ≈ 500 times faster than naive bundle adjustment.

To initialize the sparse bundle adjustment algorithm, we put all the 3D points to the XOY plane of the world coordinate system, and set all of the reference cameras at the same distance along the Z axis of the world frame, directly facing the XOY plane. The sparse bundle adjustment algorithm generally takes a few dozen iterations to converge to a reasonable solution with the above simple initialization. While requiring no knowledge of camera and scene parameters beforehand, the direct bundle adjustment approach can deal with scenes of arbitrary geometry and robustly handle noisy measurements and missing correspondences.

## 4. Natural Features Tracking and Camera Pose Computing

In the proposed framework, the camera initialization and online tracking are separated as two individual tasks. To get the feature matches in the first frame, we adopt the work of Lepetit *et al.* [29] in which wide-baseline feature matching is formulated as a classification problem. Each feature of the scene selected during the offline stage is considered as a class corresponding to the set of all its possible appearances simulated using affine transformation. The generated view set of the selected features are used to build the randomized trees. At each internal node, a set of simple tests involving intensity comparison between two pixels are randomly drawn. At each leaf node, the number of reached patches of each feature class is stored. This is an estimate of the conditional distribution over the classes given that a feature reaches that leaf. To improve matching performance, multiple randomized trees are trained independently. During the matching phase, an input feature is dropped down each tree independently. The class which has the maximum average patch number amongst those stored in all reached leaf nodes is returned as the matching result.

However, we find that the above method cannot provide satisfactory performance for non-planar structures. This is mainly because the affine transformation cannot simulate the projective deformations of non planar structures very well. To overcome the above problems, we implement the following two improvements to the work of Lepetit.

Firstly, since we have reconstructed the 3D position of each matched features in advance, we use projective transformation together with homography to generate the needed patches used to build the classification trees. Compared with affine transformation, our method can obtain better simulating performance by stressing local transmutations. We first generate some random camera positions surrounding the first reference camera (All the generated cameras' optical axis point to the barycenter of the recovered 3D points set), and then use the generated cameras (projective matrix P) to transform the feature point and its three nearest features to the simulated image spaces and calculate the homographies as follows:

(11) $$\left[\begin{array}{c} x_{s}\\ y_{s}\\ 1 \end{array}\right]∼\text{H}\left[\begin{array}{c} x_{r}\\ y_{r}\\ 1 \end{array}\right]$$

where x_r_ = (*x_r_, y_r_*, 1)*^T^* is the feature point on the reference image. x_s_ = (*x_s_, y_s_*, 1)*^T^* is the feature point on the transformed image. **H** can be calculated using four pairs of correspondences and SVD method. We use the above homographies to transform the feature's neighboring pixels to the target spaces to simulate the changes of the local patches. The generated patches are used to construct the classification trees which will be used for system initialization.

**Figure 1.**
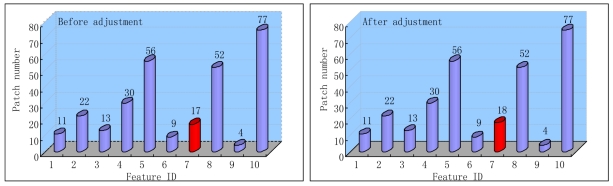
Update of the patch number of a leaf node. The figure is for illustration purposes. A usable system contains larger numbers of features and training patches.

Secondly, we also adjust the patch numbers of each tree's leaf nodes according to the tracked and recovered features. The system initialization performance is not only determined by the offline training process, but also can be ameliorated by the online tracking states dynamically. Figure 1 gives a simple illustration of the adjustment process. For each input frame, the tracked and recovered features are used to reinforce the distributions on the reached leaf nodes by increasing the patch numbers of the feature classes corresponding to them. We only update the leaf nodes of the trees while leaving the internal nodes and the structure of the trees intact. We also take two measures to preserve the diversity and validity of the classification trees. First, we only adjust the trees when the camera pose is estimated correctly. Second, we also select some key positions on the camera trajectory. The adjustment is only carried out when the difference between the current position and the selected ones is larger than the predefined thresholds. The above adjustment is implemented as an individual auxiliary thread, and will not deteriorate the processing time of the online tracking and augmentation obviously.

**Figure 2.**
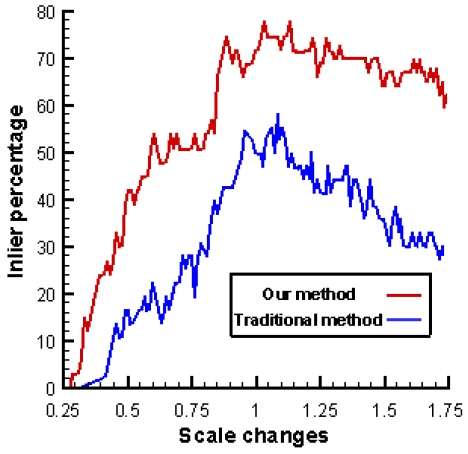
Influence of the proposed method.

Figure 2 shows the percentage of inlier matches as a function of the distance between the camera and the object, assuming this distance is 1 for the training image. In this experiment, we select 137 features and generate 1,000 patches using random projective transformations for each feature. A forest of 30 trees is used to organize the generated patches. We can see that when actual frames are used to adapt the trees, the matching performances are significantly improved which clearly shows the validity of the proposed method.

With the feature matches obtained, we can now compute the initial pose for tracking use. We use *T_d,d_* test [36,37] to speed up the outliers removal process. A time limit is also set for the *T_d,d_* test algorithm. This ensures that, in cases when the camera is occluded for a short period of time, new frames are periodically tried. A time limit of 100 ms is chosen, which is felt to be enough time to try a reasonable number of candidates. If the time limit is much higher, the result may be too far out of date when found. With the innerlies set obtained, the obtained camera pose is then optimized by minimizing the actual residual errors given as follows:

(12) $$\sum_{j=1}^{n}\left\|{\text{x}}_{j}-\xi ([\text{R}|\text{t}],{\text{X}}_{j})\right\|$$

Once the initialization has been completed, the next step is to track features between consecutive frames using narrow baseline techniques. An ideal feature tracker should cope with temporal occlusion and be able to continue to track a feature, even if it moves out of the image and returns back into the image. Therefore a feature must not be discarded, even if the feature moves out of the image.

The scale invariant feature transform gives us the chance to address the above problems. It operates in following steps [23]:

1. Search over all scales and image locations to identify potential interest points that are invariant to scale and orientation change.
2. Determine the location and scale at each candidate location; select the keypoint based on measures of their stability.
3. Assign one or more orientations to each keypoint based on local image gradient directions.
4. Generate keypoint descriptor by measuring local image gradients at the selected scale in the region around each keypoint.

For matching, features are first extracted from the input image and transformed relative to the orientation and scale. The transformed feature is compared with each feature from the reference image to find candidate matching features based on Euclidean distance of their feature vectors.

We can see that SIFT has two very important properties that can be used to solve the problem of feature loss in traditional optical flow trackers. Firstly, SIFT provides a descriptor for each feature. This property gives us the chance to recover the lost features by searching in the candidate regions using Euclidean distance based matching technique. Secondly, feature points are detected in different resolutions and transformed to the assigned orientation before matching. That is to say, we can get back the features even under large scale and viewpoint changes.

However, we cannot use SIFT directly because their primary intention is for off-line object recognition and computational demands prohibit their usability for real-time AR systems. To speed up the algorithm, we employ two important differences to that used in the SIFT. First, feature points are detected using a fast saliency operator [38]. This avoids the computationally expensive task of constructing scale space representations for each input frame. Second, scale invariance is done by constructing descriptors over multiple resolutions for each feature in offline stage. Crucially, the current registration matrix is used to guide feature and resolution matching during online tracking, leading to fast and reliable recovery.

The projective transformations similar to the method used in building classification trees are taken to generate descriptors of the matched features corresponding to different resolutions and view angles. The mainly differences are as follows: Firstly, the generated cameras do not need to point to the baricenter of the recovered 3D points set. Secondly, the rotations around the Z-axis are removed since these are accommodated for by compensating for dominant orientation when generating the descriptors. Finally the parameters used in projective transformations are generated regularly instead of randomly.

In online tracking, optical flow tracker is used to get the feature correspondences by which we can calculate the needed registration matrix. For feature recovering, we search for the best matching in the immediate neighborhood of the reprojection position of the lost feature. Since there may be some errors in pose estimation, we define a search region for the feature searching. This range is a function of the error caused by the offline reconstruction error *err_recon_* and the error coming from the pose estimation *err_pose_*. Let the error functions for both coordinates are:

(13) $${\text{F}}_{x}\left({\text{err}}_{\text{recon}},{\text{err}}_{\text{pose}}\right)$$

and:

(14) $${\text{F}}_{y}({\text{err}}_{\text{recon}},{\text{err}}_{\text{pose}})$$

The search is carried out within a rectangular region defined by (*x_l_*-F*_x_, x_l_*+F*_x_, y_l_*-F*_y_, y_l_*+F*_y_*). The values of F*_x_* and F*_y_* are dynamically set to be proportional to the sum of the maximum offline reconstruction and back-projection errors. Within the search range, we pick up the most salient feature points as the candidates for recovering. The Euclidean distances between the candidates' descriptors and the lost feature's descriptor are computed to regain the lost features.

We also make use of the estimated camera to speed up our feature recovering step. Firstly, the translation vector t = [*t_x_, t_y_, t_z_*] of the estimated camera is used to limit the search candidates by find the generated descriptors which is close to the current position. With the selected descriptors, we further limit the candidates using the current rotation vector R = [*r_x_, r_y_, r_z_*] by searching the descriptors which have similar rotations. When we find the closest descriptor we check if the Euclidean distance is below some predefined threshold. If it is not, we consider this feature is occluded and simply discard it.

While adjusting the classification trees, we also update the descriptors corresponding to the tracked and recovered features to improve the matching capability in the case of feature losing. The updating is only limited to the descriptors which are most close to the current camera, thus it is fast enough for online implementation.

With the natural features tracked and recovered, we directly apply the standard three-point RANSAC [39] to compute the camera pose consistent with the most matches. Then all the innerlies and Equation (12) are used to optimize the obtained camera pose for augmentation use.

## 5. Registration Algorithm

**Figure 3.**
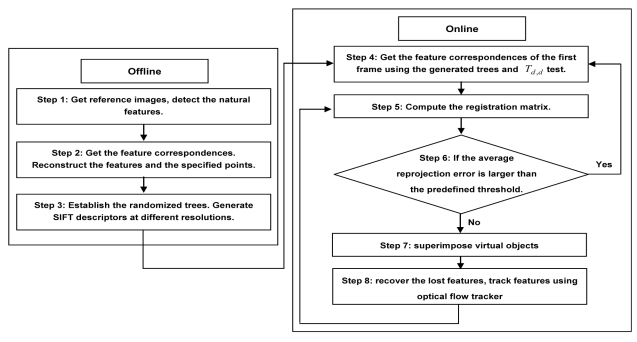
Flowchart of the proposed registration method.

This section gives the detailed descriptions of the proposed registration algorithm. Figure 3 illustrates the workflow of the proposed registration method which can be divided into two parts: scene reconstruction and camera tracking.

To reconstruct the scene structure, two control images are first selected with the camera placed at different positions. A high quality set of feature correspondences are obtained by using normalized cross-correlation operation and the eight-point RANSAC algorithm [40]. With the obtained matches, we get the camera parameters and scene structure simultaneously by using the direct bundle adjustment method discussed in Section 3. Then, four coplanar points are specified in each of the two reference frames as correspondences respectively to define the world coordinate system on which the virtual objects will be augmented. The reconstructed natural features are transformed to the established world coordinate system for online use. Finally, random projective transformation and homography are used to create the view sets to establish the classification trees which will be needed for system initialization, and SIFT descriptors at different resolutions and view angles are generated for the use of recovering the lost features in online tracking stage.

To initialize the system, we use the established classification trees to obtain the feature correspondences between the reference and input frames. Then the *T_d,d_* test is taken to get rid of outliers, after which the Equation 12 is used to calculate a precise solution to initialize the tracking system. If a valid pose cannot be obtained within the predefined time limit, a new frame is fed and the initialization process is repeated to keep the continuity of the system. After camera initialization, the SIFT based optical flow tracker discussed in Section 4 is used to track features between consecutive frames. The estimated camera pose are used to define the search region to recover the natural features that have been lost. The recovered natural features will be fed into the feature tracker. Hence, there will always be a sufficient number of natural features that has been tracked for the estimation of the corresponding camera pose. Both the tracked and recovered features are used to adjust the classification trees to make them more robust to the view point changes for system reinitialization.

The complete algorithm is described as follows:

Step 1: Select two images of the scene as references, detect the natural features using fast corner detector.
Step 2: Get the correct feature matches between the two selected images by repeatedly using the cross-correlation operation and the epipolar constraint. Calculate the camera parameters and the scene structure simultaneously using the direct bundle adjustment method discussed in Section 3.
Step 3: Create the view sets to establish the randomized trees. Generate SIFT descriptors of the natural features at different resolutions.
Step 4: Get the feature correspondences between the first and reference images using the generated randomized trees and the *T_d,d_* test algorithm.
Step 5: Compute the registration matrix of the current frame by the obtained feature correspondences.
Step 6: If the average reprojection error is larger than the predefined threshold (3 pixels in our case), go back to step 4, otherwise, turn to the next step.
Step 7: Superimpose virtual objects using the calculated registration matrix.
Step 8: Recover the lost features, adjust trees and SIFT descriptors. Obtain the corresponding natural features between the next and reference frames using optical flow tracker and RANSAC and turn back to the step 5.

## 6. Experimental Results

This section gives the experimental results to prove the validity of the proposed registration method. All the experiments are carried out on a desktop PC with an Intel Core 2 Duo 3.0 GHz processor. Software is written in C++ using the OpenCV library. The video sequences are captured using a Logitech Pro5000 camera. The image size is 320 × 240. The camera's intrinsic parameters are solved in advance by using the GML [41] camera calibration toolbox. Readers can obtained detailed experiment results including some codes and augmented video sequences by contacting the authors.

### 6.1. Indoor Experiments

In the first indoor experiment, two reference images are taken with the camera placed at different positions. We use fast corner detector to find the natural features in the two selected images. Next, the feature correspondences are obtained using the normalized cross-correlation and the eight-point RANSAC algorithm. Relative orientation for the two views is next computed by using direct bundle adjustment method discussed in Section 3. Four pairs of matched points are specified in the two reference images. The 3D coordinates of the natural features and the four specified points are computed. Then, the 3D points are transformed to the world coordinate system defined with the four specified points. For each feature correspondence, we construct a view set made of 1,000 samples using the first reference image, synthesized from projective transformations with *θ_z_* ∈ (−90,90) degrees, *t_x_, t_y_* ∈ (−2*T*,2*T*), *t_z_* ∈ (*T*/2,2*T*) where *T* is the maximal value of the second reference camera's translate vector. All the generated cameras' optical axis point to the barycenter of the recovered 3D points set. Twenty classification trees are established using the generated view sets. SIFT descriptors are built regularly using projective transformations with *θ_x_, θ_y_* ∈ (−60,60) degrees, *t_x_, t_y_* ∈ (−5*T*,5*T*), *t_z_* ∈ (*T*/4,3*T*). The changing steps are 10° and *T/4* for *θ* and *t* respectively. We use patch sizes of 22 × 22 and 4 × 4 histograms, giving descriptors with 128 elements. Using the method discussed in Section 5, the registration matrices are computed during the online registration process.

**Figure 4.**
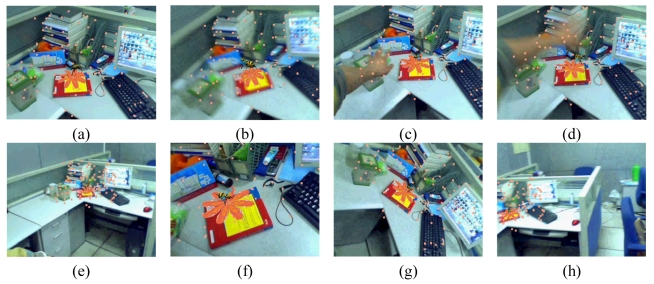
Results of the first indoor experiment.

Some augmented images are shown in Figure 4. Figure 4a gives the results when camera moves smoothly. Figure 4b gives the results when moving camera quickly. Figures 4c,d are the results in the case of occlusions. Figures 4e,f give the results when the camera is moving along the Z-axis with significant (4–0.4 times the depth of the reference image) scale changes. We can see that some of the natural features have been moved out of the visual field. However, due to the proposed recovery method, the lost features can be regained when they return to the image again. Thus, we always have a sufficient number of natural features to estimate the corresponding registration matrix. Figure 4g is the results with viewing angle changes. Figure 4h shows the results under the illumination changes. We can see that the virtual object can be augmented accurately under all the above cases. These results demonstrate the validity of the proposed registration method.

**Figure 5.**
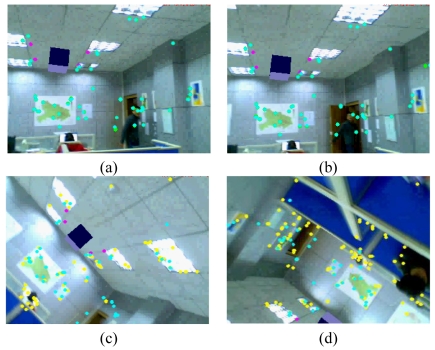
Results of the second indoor experiment.

The second experiment is used to validate the performance of the adaptive classification trees. From Figure 5, we can see that with the online dynamic adjusting, the system can recover from failures even when current cameras (Figures 5c,d) are different from the reference poses (Figures 5a,b) to a large degree (the yellow points are the detected features and the green points are the inliers used to initial the tracking systems).

**Figure 6.**
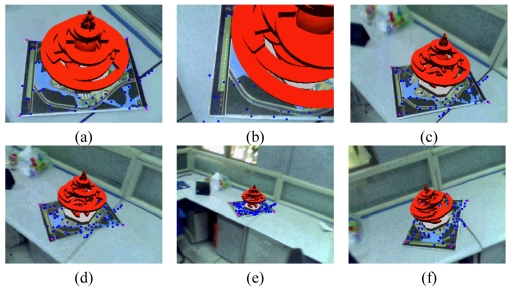
Results with planar scene.

We also take an experiment to proof the validity of the proposed method for planar structures. Figure 6 gives the results of our method under planar structure. In this experiment, cameras have been moved through large changes of viewing angles and volumes. However, due to the reduced SIFT based optical flow tracker, we can superimpose the virtual word successfully under the above circumstances.

### 6.2. Outdoor Experiments

**Figure 7.**
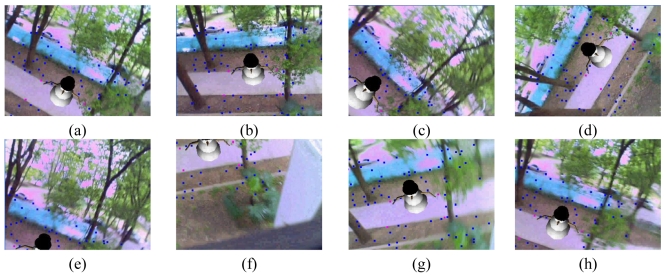
Results of the outdoor experiment.

We also performed an experiment to validate the usability of the proposed method for outdoor AR applications. We use a virtual snowman model in this experiment. Figure 7 gives the results of the experiment together with the tracked natural features. Figure 7a gives the results when camera moves with normal speed. Figure 7b shows the results under the illumination changes. Figures 7c–f give the results under large changes of the viewing angles and volumes. Figures 7g,h show the results when suddenly moving. The virtual snowman can be augmented precisely under all the above cases. These results demonstrate the validity of the proposed registration method for outdoor environments.

### 6.3. Compute Time and Feature Recover Performance

**Figure 8.**
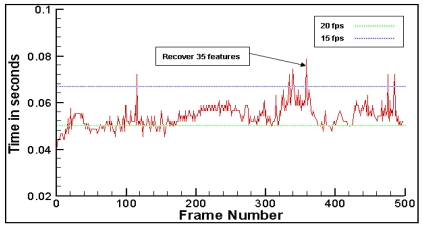
Computation time.

**Figure 9.**
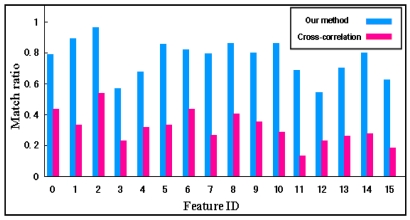
Feature recovering performance.

The system can run the proposed method at a speed of about 16 fps without the use of complicated virtual models. Figure 8 gives the processing time per frame during tracking 128 features. In this experiment, 87 feature points have been moved out of the field of view or occluded by users extremely. The processing time is always within 0.08 second which demonstrates that our method is fast enough for real time applications. We also testify the validity of the proposed feature recover method. In the experiment, 16 feature points have been occluded by user's hand frequently when moving camera. Figure 9 shows the average percentage of correct recovering for each feature for normalized cross-correlation method [8,9] and our method. Note that recovery performance is significantly better when the proposed method is used.

### 6.4. Tracking Accuracy

**Figure 10.**
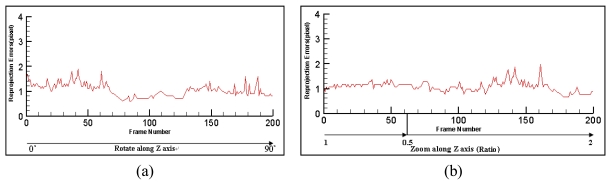
Reprojection errors.

The reprojection errors [13,14] between the original specified points and their reprojections are compared. In our experiments, the four corners of the man-made marker are used as the specified points. While tracking, the corners of the marker are detected using the ARTOOlKIT library, and their image coordinates are used as the ground truth for comparison. We are especially interested in the reprojection errors under the circumstance of large changes in rotations and zooming ratio. Figure 10a gives the reprojection errors of the proposed method when camera rotates along Z-axis from 0° to 90°. The purpose of this experiment is to simulate the case when users make large changes in view angles. Figure 10b gives the reprojection errors when camera dollies. The purpose is to simulate the case when users move close to or far from the scene. All the above errors are below 2 pixels, which demonstrates the accuracy of the proposed method.

### 6.5. Comparison with Previous Work

**Figure 11.**
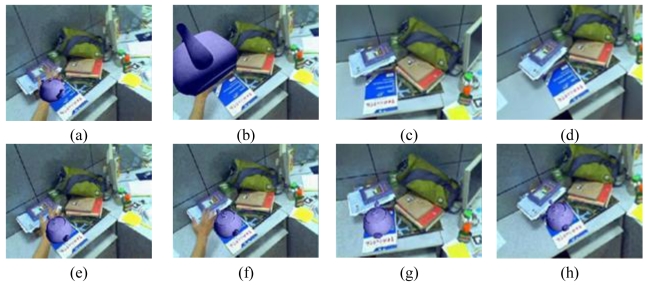
Comparison with KLT and projective reconstruction based method.

**Figure 12.**
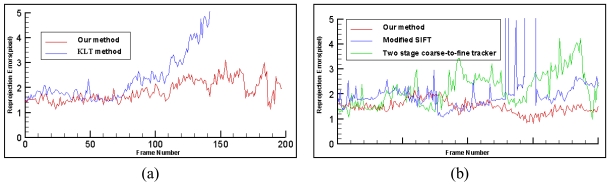
Errors comparison between our method and other methods.

Two experiments have been conducted to compare the results obtained using the proposed method with previous methods. In the first experiment, we compare our method with the KLT tracker and projective reconstruction based registration method introduced in [13,14]. As shown in Figure 11. In this experiment, the four specified points are on the Chinese journal where the virtual teapot will be augmented. Figures 11a–d show the results using the method of Yuan *et al.* Figures 11e–h show the results of our method. As given in Figures 11a,b, during the tracking process, when some parts of the scene are occluded by the hand, the virtual teapot cannot be augmented accurately using the method of Yuan *et al.* However, Figures 11e,f shows that by using the proposed registration method, the virtual object can be stably augmented, even when the scene is partially occluded. Figures 11c,d shows that the Yuan's method cannot work in the case of scene cropping. Figures 11g,h show that the proposed method can operate normally even under the cropping of the scene. The reprojection errors discussed in Section 6.4 of this experiment are also given in Figure 12a. This experiment demonstrates the advantages of the method proposed in this research.

The second experiment is carried out to compare our tracking method with the modified SIFT method [28] and two stage coarse-to-fine tracker [42]. The reprojection errors are used to compare the performance of the above three methods. From Figure 12b, we can see that our method is more stable and accurate than the other two methods, which proves the validity of the proposed tracking method.

## 7. Conclusions

In this research, we propose a novel feature tracking strategy combing wide and narrow baseline matching techniques for AR systems. Experimental results prove that the proposed method applies to both indoor and outdoor AR systems and is precise enough even in the cases of partial occlusion, rapid camera movement and large changes in volumes and viewpoints. However, there are still some issues that should be further addressed in future work to improve the performance of the proposed tracking method. One disadvantage of the current system is that the line of sight is limited to the field covered by the two reference images. The users cannot browse the virtual objects in wide area environments. We will solve this problem in future research by using structure from motion technique and wide area video sequences taken in an offline stage. Another problem is that when camera is shaking acutely, the virtual object will be lost. We find that the particle filters based top-down pose tracking strategy is very robust to erratic motion [12], so we will also try to combine bottom-up and top-down method to improve the robustness of the AR systems in the future work.
